# Phase Imaging of Phosphatidylcholine Bilayer Membranes by Prodan Fluorescence

**DOI:** 10.3390/membranes12121219

**Published:** 2022-12-02

**Authors:** Nobutake Tamai, Hitoshi Matsuki, Masaki Goto

**Affiliations:** Department of Bioengineering, Division of Bioscience and Bioindustry, Graduate School of Technology, Industrial and Social Sciences, Tokushima University, 2-1 Minamijosanjima-cho, Tokushima 770-8513, Japan

**Keywords:** diacylphosphatidylcholines, Prodan (6-propiponyl-2-(*N*,*N*-dimethylamino)naphthalene), bilayer membrane, phase transition, high pressure

## Abstract

Prodan (6-propiponyl-2-(*N*,*N*-dimethylamino)naphthalene) is well known as a polarity-sensitive fluorescent probe and has a high capability of detecting structural changes occurring within phospholipid bilayer membranes. In this study, we carried out the fluorescence spectroscopic observation of bilayer phase behavior for a series of symmetric saturated diacylphosphatidylcholines (C*n*PCs) with different acyl-chain length *n* (*n* = 12–15 and 19–22) using Prodan as a membrane probe to confirm the availability of Prodan along with the previous results for the C*n*PC bilayer membranes (*n* = 16–18). The results were discussed by constructing spectral three-dimensional (3D) imaging plots for visualizing the change in bilayer phase states with temperature or pressure to verify the functionality of this 3D imaging plot. It was found that the Prodan fluorescence technique is applicable to the detection of the changes in the bilayer phase states of all C*n*PCs with a few exceptions. One of the most crucial exceptions was that Prodan cannot be used for the detection of the bilayer-gel state of the C21PC bilayer membrane. It was also found that it is only to the C*n*PC bilayer membranes with *n* = 15–18 that the 3D imaging plot is adequately and accurately applicable as a useful graphical tool for visually detecting the bilayer phase states. This is a disadvantageous feature of this technique brought about by the high sensitivity of Prodan as a membrane probe. Further detailed studies on the molecular behavior of Prodan will enable us to find a more useful way of utilizing this membrane probe.

## 1. Introduction

Phospholipids are amphiphilic molecules and they can spontaneously form molecular aggregates with bilayer structure in the presence of excess water. Since this molecular aggregate of phospholipid bilayer membrane corresponds to the fundamental architecture of cell membranes, the structure and physicochemical properties of various kinds of phospholipid bilayer membranes have been so far studied extensively to elucidate the inherent nature of the cell membrane. Dipalmitoylphosphatidylcholine (DPPC, C16PC) is one of the phospholipids which have been most frequently used in previous model membrane studies, and it has been firmly established that the C16PC bilayer exhibits several types of structural changes with increasing temperature [1]: subtransition arising from the conversion from lamellar crystal (L_c_) phase to lamellar gel (L_β_′) phase at ca. 21 °C, pretransition from the conversion from the L_β_′ phase to ripple (P_β_′) phase at ca. 34 °C, and main transition from the conversion from the P_β_′ phase to liquid crystalline (L_α_) phase at ca. 42 °C. It is also known for the C16PC bilayer that a non-bilayer structure called interdigitated gel (L_β_I) phase is induced at high pressures above ca. 100 MPa [2,3,4], in which the hydrocarbon chains of the phospholipid molecules within a monolayer constituting a bilayer penetrate alternatively into the hydrocarbon chain region of the other monolayer of the bilayer. Now we have enough knowledge about membrane properties and structure for various kinds of phospholipids to systematically explain how the variation in the chemical structure of the constituent phospholipid affects the whole membrane properties and structure.

Such systematic knowledge about phospholipid bilayer membranes provides us with the possibility of developing new lipid-based technology that allows us to make wide use of phospholipid liposomes (i.e., particles with bilayer shells and an aqueous core) as nanobiomaterials. A liposomal drug delivery system [5] is a typical example of the practical application of lipid-based technology, in which liposomes are used as drug carriers by taking advantage of a characteristic ability of liposomes to encapsulate substances inside the bilayer shell. Actually, it is well known that this technology is applied to the production of several types of mRNA vaccines against SARS-CoV-19 infection for the delivery of mRNA [6]. A large number of studies on the liposomal drug system are now still being conducted from various aspects to realize a more effective system with high functionalities, such as enhanced target selectivity and reduced side effects. For this purpose, we need to solve quite a few issues not only relevant directly to such high functionalities but also with respect to basic technology that enables us to control the size and stability of liposome particles optimally. In addition, it is also important to establish an adequate evaluation method that enables us to easily monitor the state of liposome particles in order to verify whether liposomes exist in a state where they can well function as drug carriers.

We have so far performed systematic studies on thermodynamic properties and barotropic phase behavior of bilayer membranes of various kinds of phospholipids with different chemical structure [4,7,8,9,10]. In some of those studies, the identification of the bilayer phase states was carried out by fluorescence spectroscopy using Prodan (6-propiponyl-2-(*N*,*N*-dimethylamino)naphthalene) as a probe. Prodan is a polarity-sensitive membrane probe [11,12,13] and has an advantageous feature as compared with many other fluorescent probes that have been previously reported to be applicable as membrane probes. In general, those membrane probes are classified either as hydrophilic probes or as hydrophobic ones, and each type of probes tend to be partitioned into the hydrophilic region or the hydrophobic core of the bilayer membrane. Though Prodan is generally regarded as a hydrophilic probe, it functions in practice as both hydrophilic and hydrophobic probe within phospholipid bilayer membranes due to the weak hydrophobicity imparted by the presence of a propionyl chain attached to the naphthalene ring. This dualistic nature of Prodan enables it to respond sensitively to the changes in the bilayer state that occur not only in the hydrophilic region but also in the hydrophobic region of the phospholipid bilayer membrane. As a result, we can observe many types of bilayer phase transitions by using only one kind of fluorescent probe, Prodan. This advantage is evident from the fact that another fluorescent probe Laurdan, which has the same fluorophore and a lauroyl chain instead of the propionyl chain, cannot detect the bilayer interdigitation under high pressure [14]. In addition, it has been reported that the Prodan fluorescence technique is suitably applicable to the observation of the phase behavior of cholesterol-containing binary bilayer membranes [15,16,17]. Considering such wide availability of Prodan as a membrane probe, it is quite possible that we can develop an evaluation method for easily monitoring the bilayer state of phospholipid liposome particles by applying the Prodan fluorescence spectroscopy.

Needless to say, Prodan is not an all-purpose probe and has disadvantages. For example, the above-described wide availability can be a weak point in the case where it is used to monitor a specific structural change in a bilayer membrane, because it is better to use another probe designed for that specific purpose. It is also a disadvantageous feature that since Prodan has a hydrophilic nature, a tiny amount of Prodan is distributed into the water phase [18,19,20]. It is therefore necessary to subtract the effect arising from the Prodan molecules partitioned into water phase when we need to obtain more accurate information from Prodan fluorescence spectra. In addition, a recent report by Suhaj et al. [21] has shown that the presence of Prodan molecules within a bilayer membrane affects its local environment, altering the hydration state and the order of the lipid molecules. This could impair the reliability of the Prodan fluorescence technique as an experimental technique for the structural analysis of phospholipid bilayer membranes. In order to establish an evaluation method for easily monitoring the bilayer phase state by applying the Prodan fluorescence technique, therefore, we should make it clear how crucial or trivial effects these disadvantageous features of Prodan have on the evaluation, even though scientific strictness is not so much required when it is used only for the qualitative evaluation.

In this study, we conducted the fluorescence spectroscopic observation of bilayer phase behavior for a series of symmetric saturated diacylphosphatidylcholines (C*n*PCs) with different acyl-chain length *n* (*n* = 12–15 and 19–22) with Prodan as a membrane probe to confirm the availability of Prodan along with the previous results for C*n*PCs with *n* = 16–18 [22]. The results were discussed by constructing spectral three-dimensional (3D) imaging plots for visualizing the change in bilayer phase states with temperature or pressure to verify the functionality of this 3D imaging plot. The analysis method based on this 3D imaging plot is a method that we developed for the analysis of Prodan fluorescence spectra. Several ways of analyzing the Prodan fluorescence data, such as a method based on generalized polarization and center of spectral mass [15,19], have so far been proposed, but our method has a unique feature of providing information about the bilayer phase behavior in a visual manner. By using this plot, therefore, we can understand, at a glance, how the bilayer states change with temperature or pressure. The aim of this study is to clarify the applicability of the Prodan fluorescence technique by determining the range of the acyl-chain length through the observation for the series of the C*n*PC bilayer membranes (*n* = 12–22) and also to verify the usefulness of the 3D imaging plot as a technological application for the evaluation of the bilayer states of liposome particles.

## 2. Experimental

Synthetic diacylphosphatidylcholines, C12PC (1,2-dilauroyl-*sn*-glycero-3-phosphocholine), C13PC (1,2-ditridecanoyl-*sn*-glycero-3-phosphocholine), C14PC (1,2-dimyristoyl-*sn*-glycero-3-phosphocholine), C15PC (1,2-diheptadecanoyl-*sn*-glycero-3-phosphocholine), C19PC (1,2-dinonadecanoyl-*sn*-glycero-3-phosphocholine), C20PC (1,2-dieicosanoyl-*sn*-glycero-3-phosphocholine), C21PC (1,2-diheneicosanoyl-*sn*-glycero-3-phosphocholine) and C22PC (1,2-didocosanoyl-*sn*-glycero-3-phosphocholine) were purchased from Avanti Polar Lipids (Alabaster, AL, USA) or Sigma-Aldrich Co. (St. Louis, MO, USA). The fluorescent probe of Prodan (6-propionyl-2-(*N*,*N*-dimethylamino)naphthalene) was purchased from Molecular Probes Inc. (Eugene, OR, USA). All the materials were used without further purification. Water was distilled twice from dilute alkaline permanganate solution for the preparation of sample solutions. Multilamellar vesicle (MLV) dispersions of C*n*PC were prepared by the following procedure. As a first step, stock solutions of Prodan/ethanol and C*n*PC/chloroform were individually prepared and appropriate amounts of both stock solutions were mixed. After the mixed solution was transferred to an egg-plant shaped flask, the solvent was removed completely in vacuum. Water was added to the egg-plant shaped flask to disperse the resulting dry thin film of C*n*PC containing Prodan, and finally, we obtained a homogeneously translucent MLV dispersion after vortexing and sonicating it for a few tens of seconds at a temperature a few degrees above the main-transition temperature of each C*n*PC bilayer membrane. The concentrations of the phospholipid and Prodan were fixed at 1 mmol kg^–1^ and 2 μmol kg^–1^ (i.e., molar ratio of C*n*PC to Prodan is 500:1), respectively, for each aqueous dispersion of C*n*PC MLV.

Fluorescence measurements were performed using an F-2500 fluorescence spectrophotometer (Hitachi High-Technology Corp., Tokyo, Japan) equipped with a PCI-400 high-pressure cell assembly with sapphire windows (Syn Corp. Ltd., Kyoto, Japan). Pressure was generated by a hand-operated KP-3B hydraulic pump (Hikari High Pressure Instruments, Hiroshima, Japan) and was monitored by using a Heise gauge within the accuracy of 0.2 MPa. The temperature of a sample solution in the high-pressure cell was controlled within the accuracy of 0.1 °C by circulating water from a water bath through the jacket enclosing the sample cell. Fluorescence spectra of Prodan in C*n*PC bilayer membranes were obtained at every 1 °C under high pressure by heating a sample solution at a constant rate (0.50 °C min^–1^) within a suitable temperature range for the observation of the bilayer phase behavior of each C*n*PC. The excitation wavelength was 361 nm and emission spectra were recorded within the wavelength range from 400 nm to 600 nm. The second derivatives of emission spectra were obtained by using FL-solutions software attached to the apparatus and Origin 8.1 (Lightstone Co., Tokyo, Japan). A spectral three-dimensional imaging plot for each C*n*PC bilayer membrane was constructed by stacking all the second derivative spectra obtained at every 1 °C using SigmaPlot 12.5 (Systat Software Inc., Palo Alto, CA, USA).

## 3. Results and Discussion

### 3.1. Second Derivative of Prodan Fluorescence Spectra and 3D Imaging Plot for C17PC Bilayer Membrane [9,22]

**Figure 1:**
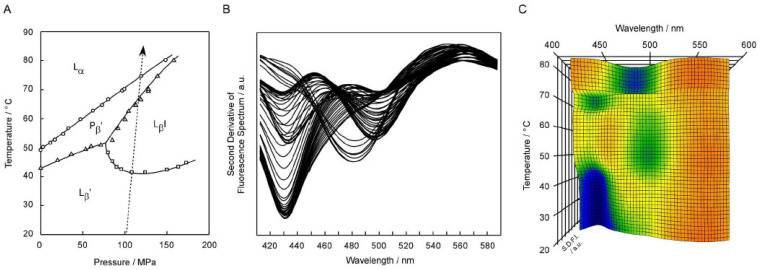
(**A**) Temperature (*T*)–pressure (*P*) phase diagram of the C17PC bilayer membrane. (**B**) Second derivatives (∂^2^*F*(λ)/∂λ^2^) of the original spectra *F*(λ) obtained directly from Prodan fluorescence measurements for C17PC bilayer membrane. (**C**) Spectral 3D imaging plot for C17PC bilayer membrane constructed by stacking all the second derivative spectra shown in (**B**). This figure was reproduced with some modification from our previous studies [9,22].

In the first place, we simply explain how we observe the bilayer phase behavior of phospholipids on the basis of the Prodan fluorescence spectroscopy and how to construct 3D imaging plots from the results of a series of fluorescence spectra obtained. This technique is basically based on the fact that the wavelength λ_max_, at which the emission maximum is observed, depends on the polarity of the environment around the Prodan molecule [12,13]. Since the microscopic polarity within phospholipid bilayer membranes steeply decreases along the bilayer depth direction from the hydrophilic surface to the hydrophobic core [23], the λ_max_ value changes definitely when the location of the Prodan molecules incorporated into a phospholipid bilayer membrane changes slightly in the depth direction with the variation in the packing state due to bilayer phase transitions. More detailed explanation about the relation between the location of Prodan molecule and the bilayer phase states has been given in our previous reports [14,22]. As an example, Figure 1 shows the results of the Prodan fluorescence spectra obtained under high pressure (ca. 106–117 MPa) for the C17PC bilayer membrane at every 1 °C in the range from 20 °C to 85 °C. In Figure 1A, the temperature (*T*)–pressure (*P*) phase diagram of the C17PC bilayer membrane [9,22] is given to indicate the heating process (the arrow in the figure) along which the fluorescence spectroscopic observation was performed. As seen from this figure, all the different four phases, including the pressure-induced L_β_I phase, are observed in this heating process. Figure 1B shows the second derivatives (∂^2^*F*(λ)/∂λ^2^) of the original spectra *F*(λ) obtained directly from the Prodan fluorescence measurements. The reason we use the second derivative spectra, and not the original spectra, is that emission peaks, including minor peaks if any, are seemingly signalized as compared to those in the original spectra. It should be noted that λ_min_ is mathematically equivalent to the wavelength λ_max_ at which fluorescence intensity becomes maximum in the original spectrum. Although this figure is not clear enough to readily grasp how the second derivative spectra change with increasing temperature, this result demonstrates that each spectrum has the minimum either at ca. 430 nm, at ca. 480nm, or at ca. 500 nm, and that the change in the wavelength λ_min_ at which the intensity of the second derivative spectrum (SDFI) becomes minimum well correlates with the change in the bilayer phase state with increasing temperature. This means that the second derivative spectra exhibit λ_min_ characteristic of the respective bilayer phase states: 430–440 nm for L_β_′ and P_β_′ phase, 480–490 nm for the L_α_ phase and ca. 500 nm for the L_β_I phase. Accordingly, it is possible for us to detect the bilayer phase transitions from the change in λ_min_.

As mentioned above, it is rather difficult to exactly understand the phase behavior of the C17PC bilayer membrane directly from Figure 1B, although it contains sufficient information necessary for that purpose. This may not be a purely scientific problem, but is not trivial in practice. Then, we introduced a 3D plot by adding a temperature axis to represent the change in the second derivative spectra with temperature more clearly. Figure 1C shows the 3D plot thus constructed for the C17PC bilayer membrane by stacking all the second derivative spectra shown in Figure 1B. In this 3D plot, the wavelength axis and the temperature axis are set horizontally and vertically, respectively, and the fluorescence intensity (i.e., SDFI) is indicated by color from blue (lowest) to red (highest) to depict the intensity in the direction of height (i.e., normal to this sheet plane). Since the λ_min_ value is significant in the Prodan fluorescence spectroscopic observation of the phospholipid bilayer membranes, as described above, we can easily understand how the bilayer phase state changes with temperature, by locating the blue region (or green region when the minimum is not low enough) in the 3D plot. For example, it can be easily understood from Figure 1C that the C17PC bilayer membrane exists in the L_β_′ state below ca. 42 °C and the phase state changes to the L_β_I state above that temperature up to ca. 65 °C, because it can be seen from this 3D plot that below ca. 42 °C a blue region is located about 430 nm, indicative of the L_β_′ phase, while a green region is around ca. 500 nm, indicative of the L_β_I phase, between ca. 42 °C and ca. 65 °C. We call this type of plot “3D imaging plot”, because it provides us with the information in a visual manner, as is similar to general imaging technology.

### 3.2. Applicability of Prodan Fluorescence Spectroscopy to CnPC Bilayer Membranes

In a previous study [22], we have reported that the Prodan fluorescence spectroscopy based on this 3D imaging plot can be applied to the observation of the bilayer phase behavior of C16PC, C17PC and C18PC. However, we do not have enough data to prove that this technique is also applicable to other bilayer membranes of C*n*PC with shorter or longer acyl chains. Since the hydrophobic interaction between the acyl chains of the adjacent C*n*PC molecules is enhanced by the elongation of the acyl chain, it is expected that the molecular packing will become tighter with increasing in the acyl chain length. Taking into account the fact that Prodan is so sensitive as a membrane probe that it can detect the difference in the packing state arising from the difference in the curvature of the membrane [24,25], it would be probable that the change in the packing state arising from an increase or a decrease in the acyl chain length may produce an undesirable effect on the behavior of the Prodan molecules within the bilayer membrane, which may more or less impair the applicability of the Prodan fluorescence spectroscopic observation. Therefore, it is important not only for the practical application but also for scientific data to confirm the availability of this technique experimentally.

**Figure 2:**
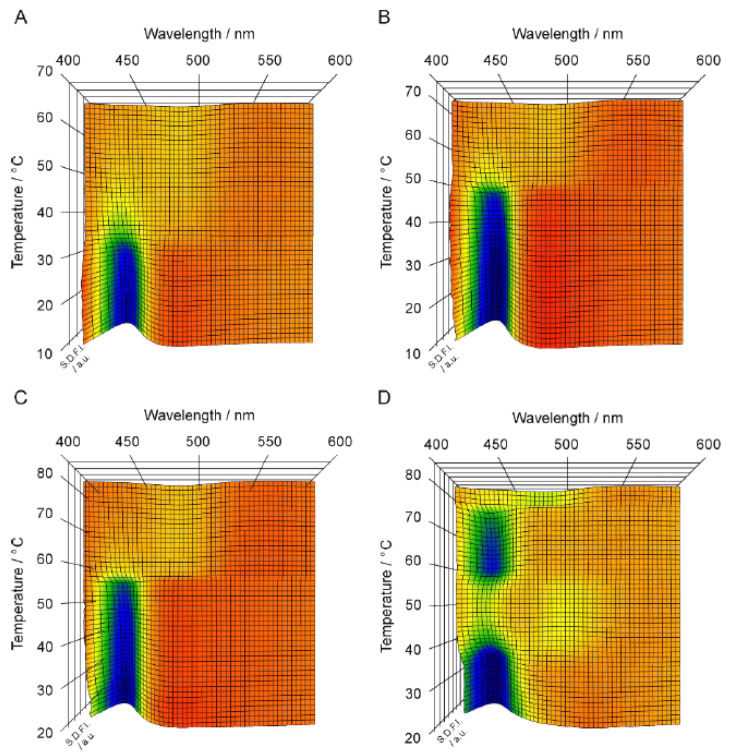
Spectral 3D imaging plots for C*n*PC bilayer membranes (*n* = 12–15): (**A**) C12PC, (**B**) C13PC, (**C**) C14PC, (**D**) C15PC. The heating process along which the Prodan fluorescence spectroscopic observation was carried out to construct each 3D imaging plot is given as an arrow shown in Figure 3.

**Figure 3:**
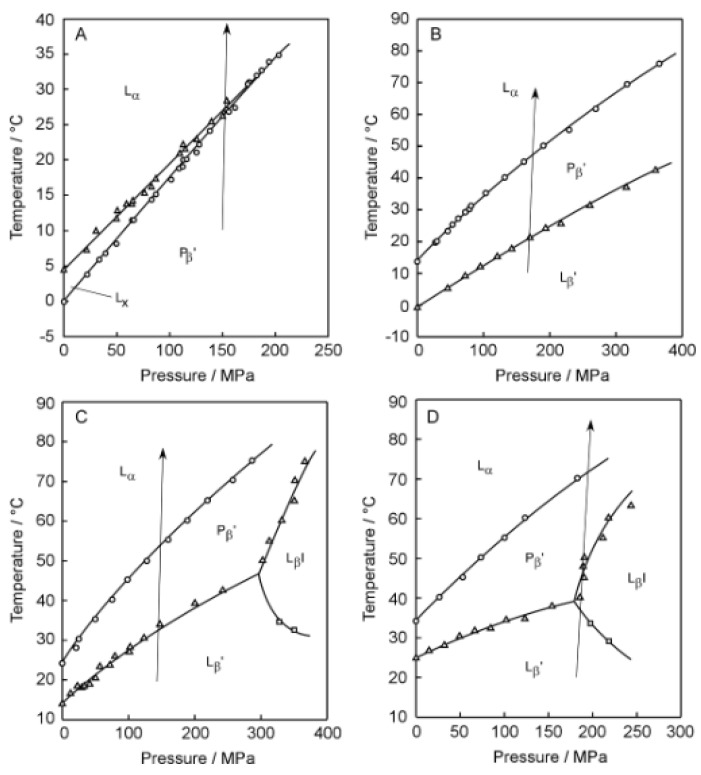
Temperature (*T*)–pressure (*P*) phase diagrams of the C*n*PC bilayer membranes (*n* = 12–15): (**A**) C12PC, (**B**) C13PC, (**C**) C14PC, (**D**) C15PC. The arrow shown in each diagram indicates the heating process along which the Prodan fluorescence spectroscopic observation was carried out to construct each 3D imaging plot shown in Figure 2.

Figure 2 shows the 3D imaging plot (Figure 2A–D) constructed on the basis of the Prodan fluorescence spectroscopic observation for the C*n*PC bilayer membrane with shorter acyl chains (i.e., *n* = 12–15). In Figure 3, the *T*–*P* phase diagrams of the corresponding bilayer membranes are given to indicate the heating process along which the Prodan fluorescence spectroscopic observation was carried out to construct the 3D imaging plots shown in Figure 2. Before discussing the 3D imaging plots for these C*n*PC bilayer membranes, we should explain their phase behavior under high pressure on the basis of the diagrams in brief, because the C12PC and the C13PC bilayer membrane exhibit peculiar behavior, which is somewhat different from that of the other C*n*PC bilayer membranes in the following two respects. First, the C12PC and the C13PC bilayer membrane do not form the interdigitated structure under high pressure [4,26]. As seen from the phase diagrams, this is true at least within the pressure range up to ca. 400 MPa, and there is no previous report demonstrating that the pressure-induced interdigitation occurs in these bilayer membranes above 400 MPa, as far as we know. This is probably due to relatively looser packing of the C12PC or C13PC molecules within the bilayer membrane, which is arising from the weaker hydrophobic interaction between the adjacent hydrocarbon chains. Second, the presence of another phase, generally called L_x_ phase, is detected during the process of the gel-to-liquid-crystalline transition in the C12PC bilayer membrane [26,27]. This is also true for the C13PC bilayer membrane [28], although the temperature range within which the L_x_ phase is observed is extremely narrow. The bilayer membrane in this phase exhibits intermediate structural and thermodynamic features between those in the gel phase and in the liquid crystalline phase. The details have been already described in our previous study [28] as well as in the literature cited there. These facts indicate that the packing state of the C*n*PC molecules within the bilayer membrane is altered by the variation in the acyl-chain length more markedly than we envisage.

Taking these peculiarities in the bilayer phase behavior of C*n*PC with shorter acyl chains into account, we performed the Prodan fluorescence spectroscopic measurements by setting a suitable heating process under high pressure for each C*n*PC bilayer membrane to construct a 3D imaging plot for visualizing the change in the bilayer phase state from P_β_′ to L_α_ for C12PC, from L_β_′ to P_β_′ and subsequently to L_α_ for C13PC and C14PC, and from L_β_′ to L_β_I, subsequently to P_β_′ and finally to L_α_ for C15PC. We should note that the minimum pressure required for inducing the L_β_I phase in the C14PC bilayer membrane is too high to carry out the fluorescence spectroscopic measurements, although it has already been confirmed by another optical technique applicable under such higher pressure that the interdigitation occurs in the C14PC bilayer membrane above ca. 300 MPa. For the C12PC bilayer membrane, the 3D imaging plot clearly shows the presence of a blue region around 435 nm in the temperature range below ca. 30 °C. This indicates that the C12PC bilayer membrane exists in a bilayer-gel state in this temperature range, and this is almost consistent with the phase diagram, which signifies that the phase state of the C12PC bilayer membrane below ca. 28 °C at ca. 150MPa is the P_β_′ phase. However, no blue or green region can be seen above ca. 30 °C in the 3D imaging plot, although it can barely be recognized from the color gradation that SDFI becomes minimum between 480 nm and 490 nm. From this λ_min_ value, it would be possible to presume that the C12PC bilayer membrane exists in the L_α_ phase in this temperature range. However, the fact that no blue or green region was observed means that the position of the SDFI minimum is relatively obscure. Accordingly, taking this into account, the identification of the phase state on the basis of this result alone is not as accurate as in the case of the C17PC bilayer membrane, which exhibits a definite minimum around 480 nm above ca. 75 °C as shown in Figure 1C.

Similar spectral behavior was observed in the 3D imaging plot for the C13PC and the C14PC bilayer membrane, as shown in Figure 2B,C. That is, no blue or green region is recognized in the temperature range above the main transition temperature, whereas a blue region can be clearly seen around 435 nm below the main transition temperature. This similarity suggests that the obscurity in the position of the SDFI minimum (i.e., the absence of blue or green regions) in the temperature range above the main transition temperature arises from a common feature to these C*n*PC with the relatively shorter acyl chains (i.e., *n* = 12–14). Probably, this is relevant to relatively looser molecular packing of the bilayer membrane due to the relatively weaker hydrophobic interaction between the acyl chains of the adjacent PC molecules with those shorter acyl chains. As described above in the explanation as to how the Prodan fluorescence spectroscopy works as a technique for the observation of the bilayer phase behavior, the change in the λ_min_ (or λ_max_) value with the bilayer transition is mainly due to the migration of the Prodan molecules along the bilayer depth direction within the bilayer membrane when the transition occurs. Taking this into consideration, the obscurity in the SDFI minimum can be interpreted to indicate that the Prodan molecules can move comparatively freely in the bilayer depth direction within the range between the glycerol backbone region and the hydrophilic surface region. In other words, the relatively looser molecular packing of the bilayer membrane allows the relatively free molecular motion of Prodan in the vertical direction within the bilayer membrane and as a result, the vertical distribution of the Prodan molecules becomes relatively uniform without exhibiting any sharp peak indicative of the localization of them at a specific vertical location.

In contrast to the 3D imaging plots for the C12PC, the C13PC and the C14PC bilayer membrane, the imaging plot for the C15PC bilayer membrane (Figure 2D) shows several relatively clear regions, which makes it easier for us to interpret the changes in the bilayer phase state with temperature. In particular, it is different from those for C*n*PCs (*n* = 12–14) in the respect that a greenish region was observed in the temperature range above the main transition temperature (ca. 35 °C). This difference is also explainable in terms of the change in the packing state with the elongation of the acyl chains. That is, it can be interpreted to reflect that the packing state of the bilayer membrane becomes slightly tighter with the elongation of the acyl chains by a few methylene groups. On the other hand, it still has several undesirable features with regard to the indication of the bilayer phase state. First, there is no blue or green region indicative of the L_β_I phase, though the color gradation suggests that there is a local minimum around 500 nm in the temperature range from ca. 40 °C to ca. 50 °C, where the phase diagram indicates that the C15PC membrane exists in the L_β_I phase. As seen from the 3D imaging plot, another local minimum of SDFI lies concomitantly at 433 nm (i.e., greenish region) in this temperature region, which suggests that a part of the Prodan molecules still remain in the vicinity of the glycerol backbone without being squeezed out toward the hydrophilic surface even after the bilayer interdigitation occurs. This may be relevant to the relatively looser packing state of the membrane; that is, relatively weaker hydrophobic interaction between the acyl chains enables a part of Prodan molecules to penetrate into the hydrophobic region between two retrorse acyl chains. Second, it is very difficult to discern the difference between the spectral features indicative of the L_β_′ and P_β_′ phase, although on the 3D imaging plot the two blue regions of these phases are separated by the presence of the region of the L_β_I phase. This is true also for the 3D imaging plot for the C13PC and the C14PC bilayer membrane, where it seems that there is only one blue region because the two regions of the L_β_′ and P_β_′ phase are continuous. This obscurity arises from a significant disadvantage that Prodan inherently possesses: it is not so sensitive to the change in the bilayer phase state from L_β_′ to P_β_′, which produces no or a very slight difference in the λ_min_ (or λ_max_) value (at most ca. 3 nm). Therefore, we should consider that it is virtually impossible to distinguish the bilayer phase states of L_β_′ and P_β_′ only on the basis of the 3D imaging plot. Note that this does not mean the inability of Prodan to detect the difference between the bilayer phase states of L_β_′ and P_β_′. We can determine the pretransition point correctly by taking slight differences in the fluorescence intensity and the lineshape of the spectrum into consideration to analyze the Prodan fluorescence spectra in more detail [9].

**Figure 4:**
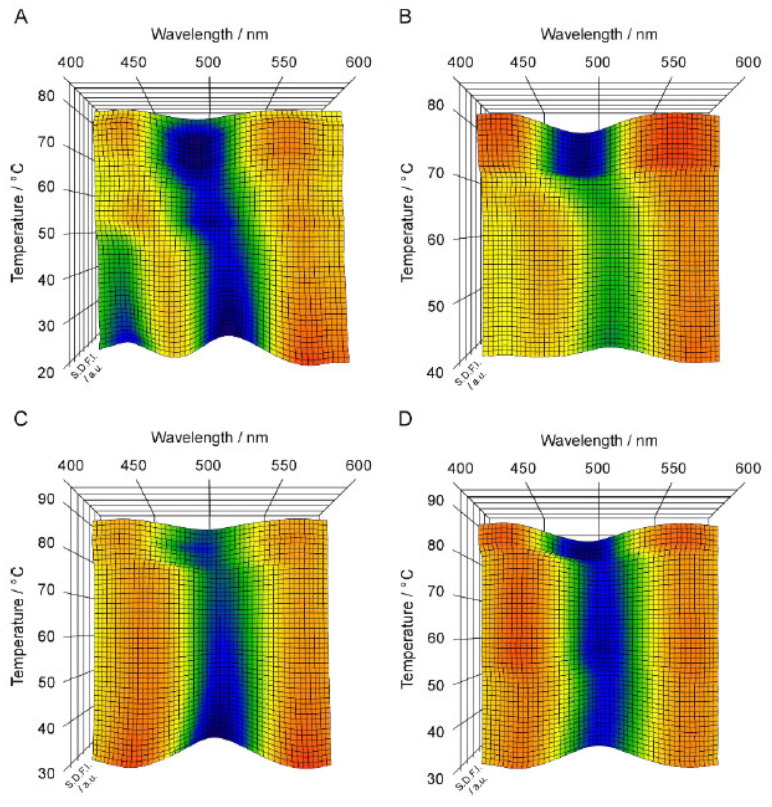
Spectral 3D imaging plots for C*n*PC bilayer membranes (*n* = 19–22): (**A**) C19PC, (**B**) C20PC, (**C**) C21PC, (**D**) C22PC. The heating process along which the Prodan fluorescence spectroscopic observation was carried out to construct each 3D imaging plot is given as an arrow shown in Figure 5.

**Figure 5:**
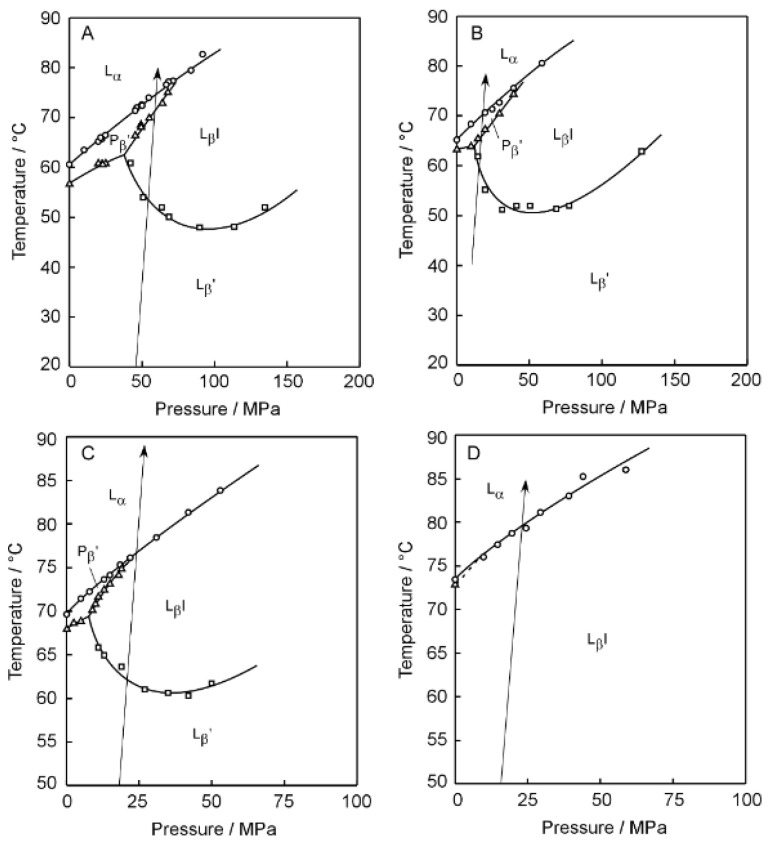
Temperature (*T*)–pressure (*P*) phase diagrams of the C*n*PC bilayer membranes (*n* = 19–22): (**A**) C19PC, (**B**) C20PC, (**C**) C21PC, (**D**) C22PC. The arrow shown in each diagram indicates the heating process along which the Prodan fluorescence spectroscopic observation was carried out to construct each 3D imaging plot shown in Figure 4.

Figure 4A–D and Figure 5A–D show the 3D imaging plots for the C*n*PC bilayer membrane with relatively longer acyl chains (i.e., *n* = 19–22) as well as the *T*–*P* phase diagrams for the indication of the heating process, respectively. Here, we should note that only the C22PC bilayer membrane exhibits peculiarly different phase behavior as compared to that of the other C*n*PC bilayers: it forms the interdigitated structure under atmospheric and high pressure at relatively lower temperatures and undergoes only one transition from the L_β_I phase to L_α_ phase in the heating process [29,30]. It should be also noted that the sequence of the phases that we observed is somewhat different because of the shrinkage of the P_β_′ phase region with the increase in the acyl chain length as follows: L_β_′, L_β_I, P_β_′ and L_α_ for C19PC and C20PC; L_β_′, L_β_I, and L_α_ for C21PC; and L_β_I and L_α_ for C22PC. As is obvious from Figure 4, the overall appearance of the 3D imaging plots for the C*n*PC bilayer membranes with longer acyl chains (*n* = 19–22) is evidently different from the results for the C*n*PC bilayer membranes with shorter acyl chains (*n* = 12–15): clear blue or green regions appear irrespective of the phase sequence in the whole temperature range measured in the imaging plots for the longer C*n*PC bilayer membranes whereas the orange area is relatively vast as a whole for the shorter C*n*PC bilayer membranes. This suggests that Prodan has a good ability to detect the change in the packing state of the bilayer membranes arising from the elongation of the acyl chains.

A notable feature of the 3D imaging plot for the C19PC bilayer membrane is, as seen from Figure 4A, that two local minima are observed as clear blue regions at ca. 430 nm and at ca. 510 nm in the temperature range of the L_β_′ phase. The former λ_min_ value is indicative of the L_β_′ phase, but no bilayer phase state is known to be indicated by the latter λ_min_ value. Since this λ_min_ value is apparently greater than that for the L_β_I phase (i.e., ca. 500 nm), it is thought to arise from the Prodan molecules present in a more hydrophilic environment. This means that the λ_min_ value of 510 nm indicates the possibility that the Prodan molecules are excluded to the interbilayer water phase near the bilayer surface, and thus means that it is not directly relevant to a certain specific bilayer phase state. Our previous study [22] has demonstrated that a similar tendency is observed for the C18PC bilayer membrane. However, the SDFI at ca. 510 nm was apparently lower than that at ca. 430 nm, which is definitely different from the result for the C19PC bilayer membrane. Taking this into account, the exclusion of the Prodan molecules from the bilayer membrane is considered to result from the tighter packing state of the longer-chain C*n*PC bilayer membrane; that is, the increase in the packing density brought about by the elongation of the acyl chains makes it harder for the Prodan molecules to transfer themselves into the bilayer membrane. This tendency is confirmed more markedly in the 3D imaging plots for the C20PC and the C21PC bilayer membrane, which display no blue or green region at ca. 430 nm but show a clear blue region around 510 nm in the temperature range where the bilayer membrane exists in the L_β_′ phase (Figure 4B,C). This indicates that the tighter molecular packing of the bilayer membrane due to the further increase in the acyl chain length hamper the incorporation of the Prodan molecules into the bilayer membrane more strongly, and as a result, almost all Prodan molecules are finally excluded from the bilayer membrane at the acyl chain length of 21. This means that the Prodan fluorescence spectroscopy is not suitable for the observation of the behavior of the L_β_′ and the P_β_′ phase for the longer C*n*PC bilayer membranes (*n* = 19–21).

In contrast to this disadvantageous feature, Prodan can detect the bilayer phase states of L_β_I and L_α_ sensitively for these longer C*n*PC bilayer membranes (*n* = 19–22), as is obvious from the fact that each imaging plot shown in Figure 4 clearly shows a blue or green region around 500 nm in the temperature range of the L_β_I phase and a blue region at 490–500 nm in the temperature range of L_α_ region. It is especially interesting that the imaging plots for the C19PC, the C20PC and the C21PC bilayer membrane clearly show the changes in the bilayer phase state from L_β_′ (at ca. 510 nm) to L_β_I (at ca. 500 nm) and finally to L_α_ (at ca. 490 nm) as the change in the location of the blue or green region, except for the P_β_′ phase that should appear in the middle of this heating process for the C19PC and the C20PC bilayer membrane. As described above, the blue region at ca. 510 nm indicates that almost all Prodan molecules exist in the interbilayer water phase. Therefore, this consecutive change in the location of the blue region means that the Prodan molecules moves from the outside of the bilayer membrane into the bilayer membrane to be localized at specific vertical positions for the bilayer phase states of L_β_I and L_α_, namely the hydrophilic surface region and the region near the phosphate group, when the bilayer membrane undergoes the transitions from L_β_′ to L_β_I, (P_β_′) and L_α_ in sequence. Judging from this migration behavior of the Prodan molecules, it seems reasonable to speculate that the Prodan molecules excluded from the bilayer membrane in the L_β_′ state are not dispersed randomly throughout the interbilayer water phase, but rather are localized in the vicinity of the bilayer surface, which enables the almost all Prodan molecules to move quickly into the bilayer membrane at the transition from the L_β_′ phase to the L_β_I phase. It is unfortunate that the behavior of the Prodan molecules in the bilayer membrane in the P_β_′ state was not captured by the 3D imaging plots for the C19PC and the C20PC bilayer membrane. This may be due to the fact that the temperature range where the bilayer membrane can exist in the P_β_′ state was too narrow to observe the spectral feature specific for the P_β_′ phase, but it is hardly expected that the λ_min_ value of 430–440 nm will be obtained for the P_β_′ state of the C19PC and the C20PC bilayer membrane because there is a strong tendency for the Prodan molecules to be excluded from the bilayer membrane in the gel state.

### 3.3. Acyl-Chain Length Dependence of λ_min_ Value Characteristic for Each Bilayer Phase

**Figure 6:**
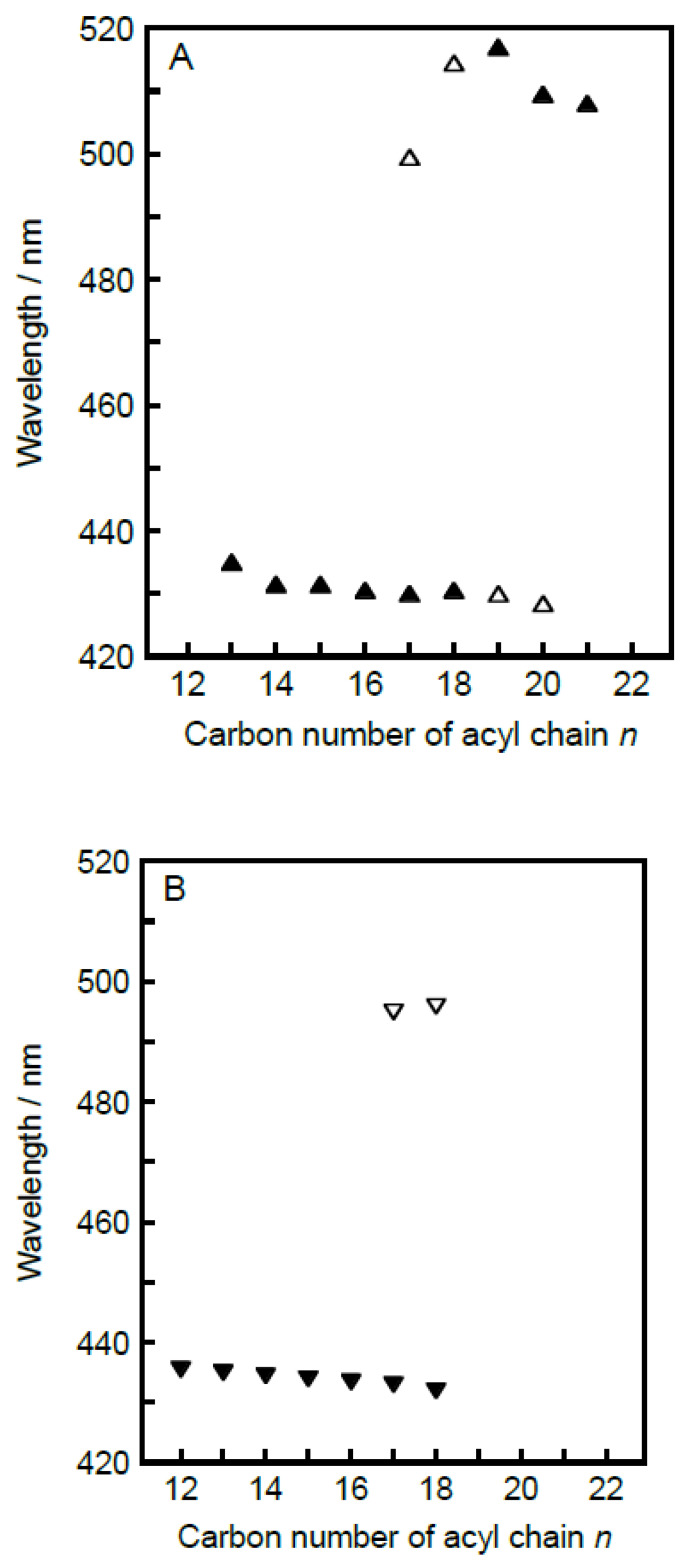

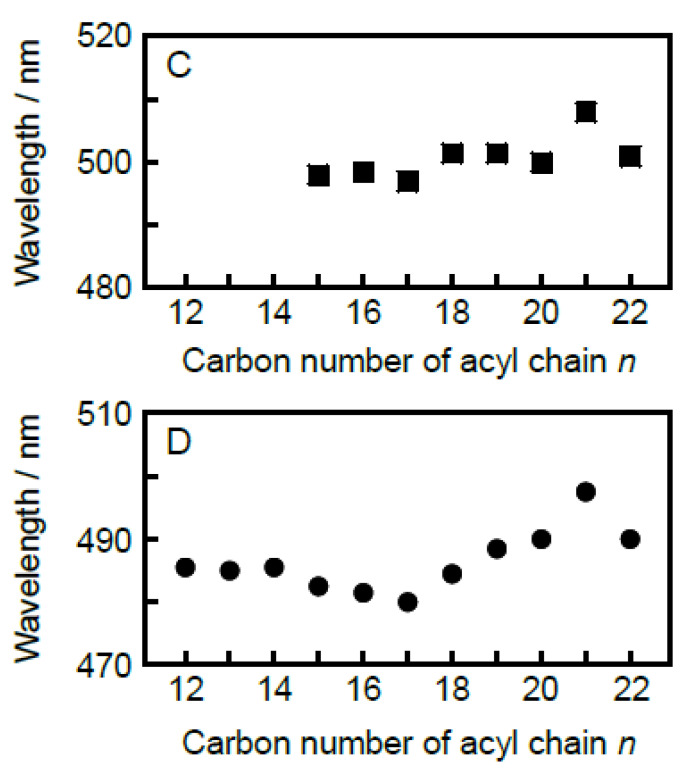
Acyl-chain length (*n*) dependence of λ_min_ caracteristics for each bilayer phase state: (**A**) lamellar gel (L_β_′) phase, (**B**) ripple gel (P_β_′) phase, (**C**) interdigitated gel (L_β_I) phase, (**D**) liquid crystalline (L_α_) phase. Here, λ_min_ means the wavelength at which a local minimum was observed in each second derivative spectrum, and open symbols are used in the pannels (**A**,**B**) to represent λ_min_ for minor peaks.

To make it clear how the spectral features that Prodan exhibits as a membrane probe change with the variation in the acyl-chain length of the constituent C*n*PC molecules, the λ_min_ values characteristic for respective bilayer phase states were plotted against the acyl-chain length *n* in Figure 6. Several rough tendencies can be seen on the whole in the acyl-chain length dependence of the λ_min_ values, which provides us with significant information about the applicability of the Prodan fluorescence spectroscopy to the observation of the bilayer phase behavior of C*n*PCs. First, we look at the λ_min_ behavior relating to the bilayer gel phases, shown in Figure 6A,B. In many of our previous studies using the Prodan fluorescence technique [9,13,14,17,22,24,25], we have regarded the λ_min_ (or λ_max_) value of 430–440 nm as a wavelength characteristic for the bilayer phase state of L_β_′, L_β_ or P_β_′(i.e., bilayer gel state). Figure 6A,B demonstrates that this rule is applicable to the C*n*PC bilayer membrane in the case of *n* ≤ 20, though the λ_min_ value of ca. 430 nm was obtained from a minor peak for *n* = 19 or 20. On the other hand, this rule is not applicable to the C*n*PC bilayer membrane with *n* = 21 or 22. With regard to the latter case (i.e., *n* = 22), it is of little significance to discuss this inapplicability, because the phase is different: the C22PC bilayer membrane does not exist in the L_β_′ or P_β_′ state below the main-transition temperature but does in the L_β_I state even at the atmospheric pressure, as mentioned above. Therefore, there is no plot at *n* = 22 in Figure 6A,B. Taking this into account, we may consider that it is only for C21PC that the above rule is not valid. As described above, this invalidity is presumed to result from relatively tighter molecular packing of the C21PC bilayer membrane in the gel state: the enhancement of the hydrophobic interaction between the adjacent acyl chains with increasing acyl-chain length brings about the tighter molecular packing, which causes almost all the Prodan molecules to be excluded from the bilayer membrane. This means that the bilayer phase behavior of C21PC cannot be suitably observed by the Prodan fluorescence technique.

As a second feature, the λ_min_ value for the bilayer gel-states tends to decrease very slightly with increases in the acyl-chain length in the *n*-range of 12–20, which implies that the local environment around individual Prodan molecules becomes slightly more hydrophobic as the acyl chain increases in length. In addition, the second peak appearing as a minor peak at ca. 510 nm for *n* ≥ 17 becomes more pronounced with increasing in the acyl-chain length: it turns to the main peak at *n* = 19 and finally it only remains as a main peak at *n* = 21. These two tendencies can be explained by supposing the so-called exclusion effect, though they may seem inconsistent with each other. As already described above, the appearance of the minor peak at ca. 510 nm suggests that a certain amount of Prodan molecules are present near the bilayer surface region in the bulk water phase. Thus, the latter tendency indicates that more Prodan molecules tend to be squeezed out from the bilayer membrane as the molecular packing state of the bilayer membrane in the gel state becomes tighter with increasing acyl-chain length (*n* ≥ 17). The former tendency is also explainable in terms of the exclusion effect as follows. This exclusion effect is expected to work not only on the Prodan molecules but also on the water molecules existing around the headgroups of the C*n*PC molecules. Taking this into consideration, the exclusion effect could also cause a reduction in the number of the water molecules that can come closer to the glycerol backbone region, where most Prodan molecules are presumed to be localized within the gel-state bilayer membrane. Eventually, the local environment around the headgroup region inside the gel-state bilayer membrane would be somewhat less hydrophilic with increasing acyl-chain length.

In the last place, we discuss the behavior of Prodan molecules within the bilayer membrane in the L_α_ or the L_β_I state. As seen from Figure 6C,D, the λ_min_ (or λ_max_) value of 480–490 nm and that of ca. 500 nm can be regarded as a wavelength characteristic of the L_α_ within the *n*-range of 12–22 and of the L_β_I phase within the *n*-range of 15–22, respectively, though both λ_min_ values at *n* = 21 are somewhat high. In particular, no significant *n*-dependence of the λ_min_ value is found for the L_β_I phase (Figure 6C), suggesting that the behavior of the Prodan molecules within the membrane with the interdigitated structure is hardly affected by the change in the length of the acyl chains. This is quite reasonable, because the Prodan molecules are pushed out to the membrane surface region near the bulk water phase by the alternately interpenetrated acyl chains as the bilayer interdigitation occurs. As for the λ_min_ value for the L_α_ phase, Figure 6D suggests that the *n*-dependence of λ_min_ comprises at least two mutually opposing effects: the value decreases slightly with increasing achy-chain length up to *n* = 17, but it conversely increases with increasing achy-chain length at *n* ≥ 17. This biphasic *n*-dependence is also explainable on the basis of the same concept as is applied to speculate on the behavior of the Prodan molecules within the gel-state bilayer membrane. That is, the tighter packing state arising from the increase in the acyl-chain length will cause the reduction in the number of the water molecules around the headgroups of the C*n*PC molecules, which can give rise to the slight decrease in the λ_min_ value with increasing acyl-chain length up to *n* = 17. Meanwhile, as the acyl chain further increases in length, the exclusion effect will be also exerted on the Prodan molecules themselves, and as a result, the most probable vertical location of the Prodan molecules within the bilayer membrane shifts more or less toward the outside of the bilayer membrane (i.e., more hydrophilic region), which brings about a slight increase in the λ_min_ value. It should be noted that the original spectra, as shown in Figure 2, exhibit a comparatively broad peak with comparatively low intensity for the L_α_-state bilayer membrane of C*n*PC with relatively shorter acyl chains (i.e., *n* = 12–15). Taking this into account, it might be better for us not to emphasize the significance of the tendency for the λ_min_ value to decrease with increasing acyl-chain length in the *n*-range below 17.

It is also interesting to note that the *n* value at which the reversal in the *n*-dependence of λ_min_ for the L_α_ phase occurs agrees with the *n* value at which the minor peak at ca. 510 nm begins to be observed for the gel-state bilayer membrane. In our speculation, as already described repeatedly, these spectral features of the Prodan fluorescence arise from the same origin: Prodan molecules begin to be excluded from the bilayer membrane at *n* = 17. Taking this into account, this agreement seems to support the consistency of our speculation, but we should consider the origin of the exclusion effect more carefully. If our speculation is true, this agreement means that the Prodan molecules tend to be excluded from the bilayer membrane irrespective of the bilayer phase states, namely either L_α_ phase or L_β_′ (or P_β_′) phase, when the acyl-chain length exceeds 17. In the case of the bilayer-gel state, it is understandable that the spatial restriction due to tighter packing state with increasing acyl-chain length will cause the exclusion effect. On the other hand, considering the fact that the packing density of the bilayer membrane in the L_α_ state is intrinsically loose as compared to that of the gel-state bilayer membrane, it does not seem probable that the spatial restriction will similarly cause the exclusion effect on the Prodan molecules in the L_α_-state bilayer membrane within the same range of the acyl-chain length. However, as seen from the change in color of the L_α_ region on the 3D imaging plot with the increase in the acyl-chain length, the peak position becomes more obvious and the peak intensity becomes higher as the acyl-chain length increases, which indicates that the vertical location of the Prodan molecules within the bilayer membrane tends to be fixed. Taking this tendency into account, the packing density of the C*n*PC bilayer membrane with relatively longer acyl chains (*n* ≥ 17) may be tight enough, even in the L_α_ state, to produce the exclusion effect, when it is looked at from the viewpoint of the molecular behavior of Prodan. In order to clarify this experimentally, more detailed thermodynamic and structural investigation on the behavior of the Prodan molecules within the bilayer membrane is required.

## 4. Conclusions

In this study, we carried out the fluorescence spectroscopic observation of the bilayer phase behavior under high pressure for a series of C*n*PCs within the *n*-range of 12–22 with Prodan as a membrane probe to confirm the availability of the Prodan fluorescence technique. Except for a few exceptions, our rules for the identification of the bilayer phase states, which have been applied in our previous studies, were almost applicable for all the C*n*PC bilayer membrane investigated in this study: λ_min_ = 430–440 nm for the bilayer-gel states (i.e., L_β_, L_β_′ or P_β_′ phase), 480–490 nm for the L_α_ state and ca. 500 nm for the L_β_I state. One of the most crucial exceptions is that the Prodan fluorescence technique does not work for the detection of the bilayer-gel state of the C21PC bilayer membrane although it is possible to identify the L_α_ phase of the same bilayer membrane by this technique. This is presumed to be due to the fact that all the Prodan molecules are excluded from the inside of the bilayer membrane to the surface region outside the bilayer membrane owing to the relatively tight molecular packing.

In order to make it possible to understand the bilayer phase behavior more intuitively from the obtained spectral data, we constructed the 3D imaging plots for each C*n*PC bilayer membrane on the basis of all the second derivative spectra obtained at every 1 °C over the whole temperature range within which we performed the measurements. For the bilayer membranes of C*n*PCs with relatively shorter acyl chains (*n* = 12–14), our 3D imaging plot was not successful in detecting the L_α_ state as a blue or green region around λ_min_ = ca. 490 nm, though the bilayer-gel state was clearly recognized as a blue region on the 3D imaging plot. At *n* = 15, however, a green region indicative of the L_α_ state appeared above the main-transition temperature on the 3D imaging plot, and as the acyl chains became further longer, this L_α_-region on the 3D imaging plot became more clearly observable as a blue region. Judging from this tendency, we can conclude that only in the case of *n* ≥ 15, this 3D imaging plot is successfully applicable for the purpose of visually detecting the L_α_ state of the C*n*PC bilayer membrane. In contrast to this tendency, the C*n*PC bilayer membranes with relatively shorter acyl chains (*n* ≤ 15) exhibited a clear blue region at λ_min_ = ca. 430–440 nm, indicative of the bilayer-gel state, on the 3D imaging plot, and this blue region shifted to higher wavelengths (i.e., ca. 510 nm) as the acyl chains became longer (*n* ≥ 19). This means that as for the detection of the bilayer-gel state, this 3D imaging plot is applicable only to the bilayer membranes of C*n*PC having not so long acyl chains (*n* ≤ 18). Taking into account these results, along with the fact that the interdigitated gel (L_β_I) phase was almost clearly observed on the 3D imaging plot for all C*n*PC with *n* ≥ 15, we can finally conclude that the 3D imaging plot is adequately and accurately applicable as a useful graphical tool for visually detecting the bilayer phase states only to the C*n*PC bilayer membranes with *n* = 15–18.

In conclusion, all the results obtained in this study would indicate that our attempt to utilize the 3D imaging plot based on the Prodan fluorescence spectra for establishing a general evaluation method for easily monitoring bilayer phase states was not successful at this stage. In order to achieve this we need to make more ingenious improvements; for example, it may be a promising strategy for future study that another probe that can cover the weak points of Prodan is concurrently used along with Prodan. On the other hand, we were able to reconfirm the potential of Prodan as a membrane probe. As described above, the 3D imaging plot was found to be useful only for C*n*PCs with *n* =15–18. This is contrary to our initial expectation, but can be interpreted as a result of the fact that the sensitivity of Prodan is extremely high, as can be seen in the *n*-dependence of each λ_min_ value characteristics for each phase state. The complex behavior of λ_min_ with the change in the acyl-chain length was explainable, at least qualitatively, in terms of the molecular packing of the bilayer membrane, the vertical position of Prodan, and the molecular density of water around the headgroup. This means that Prodan can sense the very slight change in the bilayer structure brought by the increase in the acyl-chain length. In order to better understand what is this slight change in the structure of the C*n*PC bilayer membrane and also how Prodan senses it, it is required to systematically conduct further detailed studies on the molecular behavior of Prodan within the bilayer membranes of a series of C*n*PCs. This will lead to discovery of a new way of utilizing the Prodan fluorescence technique that allows us to make the best use of the potential that it possesses as a membrane probe.

## Data Availability

The datasets generated and/or analyzed during the current study are available from the corresponding author on reasonable request.
